# A Case of Pediatric Renal Cell Carcinoma With TFE3 Rearrangement Treated With Robot-Assisted Partial Nephrectomy

**DOI:** 10.7759/cureus.86815

**Published:** 2025-06-26

**Authors:** Takayuki Ohzeki, Remon Kunishige, Taiji Hayashi, Tsukasa Nishioka, Kazutoshi Fujita

**Affiliations:** 1 Department of Urology, Izumi City General Medical Center, Izumi, JPN; 2 Department of Urology, Kindai University, Sayama, JPN

**Keywords:** minimally invasive surgery, pediatric renal cell carcinoma, robot-assisted partial nephrectomy, tfe3-rearranged rcc, xp11.2 translocation

## Abstract

Renal cell carcinoma (RCC) is uncommon in the pediatric population, and the TFE3-rearranged RCC (tRCC) subtype is characterized by translocation at Xp11.2. While robot-assisted partial nephrectomy (RAPN) is established in adult RCC treatment, its use in pediatric malignancies remains limited. We report the case of an 11-year-old boy with a progressively enlarging right renal cystic mass. Contrast-enhanced computed tomography revealed an enhancing solid component. RAPN was performed using the da Vinci Xi system, with no perioperative complications. Histopathology confirmed tRCC based on morphology and immunohistochemistry despite a negative TFE3 split FISH (fluorescence in situ hybridization) result. The patient was discharged on postoperative day 6 and remained recurrence-free at 24 months. This case illustrates the feasibility and efficacy of RAPN for pediatric RCC, suggesting that with careful planning and selection, nephron-sparing surgery may be applicable in selected pediatric oncology cases.

## Introduction

Renal cell carcinoma (RCC) is uncommon in the pediatric population, accounting for less than 5% of all pediatric renal tumors. Among the subtypes, TFE3-rearranged RCC (tRCC) is characterized by chromosomal translocations involving the Xp11.2 locus affecting the TFE3 gene [1]. tRCC is the most frequent form of MiT family translocation RCCs and occurs primarily in children and young adults [2,3]. These tumors often present with aggressive features and may be resistant to conventional chemotherapy, underscoring the need for precise diagnosis and optimal surgical treatment [4,5]. Approximately 90% of pediatric renal tumors are Wilms’ tumors, followed by clear cell sarcomas and rhabdoid tumors. RCC accounts for less than 5% of all pediatric renal tumors, with tRCC being the most frequent subtype [6].

Robot-assisted partial nephrectomy (RAPN) is widely used in adult patients with localized RCC due to its favorable perioperative outcomes and renal function preservation [7]. In contrast, its application in pediatric oncology remains limited due to technical challenges, smaller working spaces, and limited published data. However, recent advancements in surgical systems and increased experience have begun to expand the indications for robotic surgery in children [8].

Here, we present a rare case of pediatric tRCC successfully managed by RAPN. To the best of our knowledge, this is the second reported case of its kind. This article has not been previously presented or published in any form, including abstract, poster, or oral presentation.

We report a rare case of pediatric tRCC successfully treated with RAPN and discuss its technical feasibility, perioperative outcomes, and diagnostic considerations.

## Case presentation

Patient details

**Figure 1:**
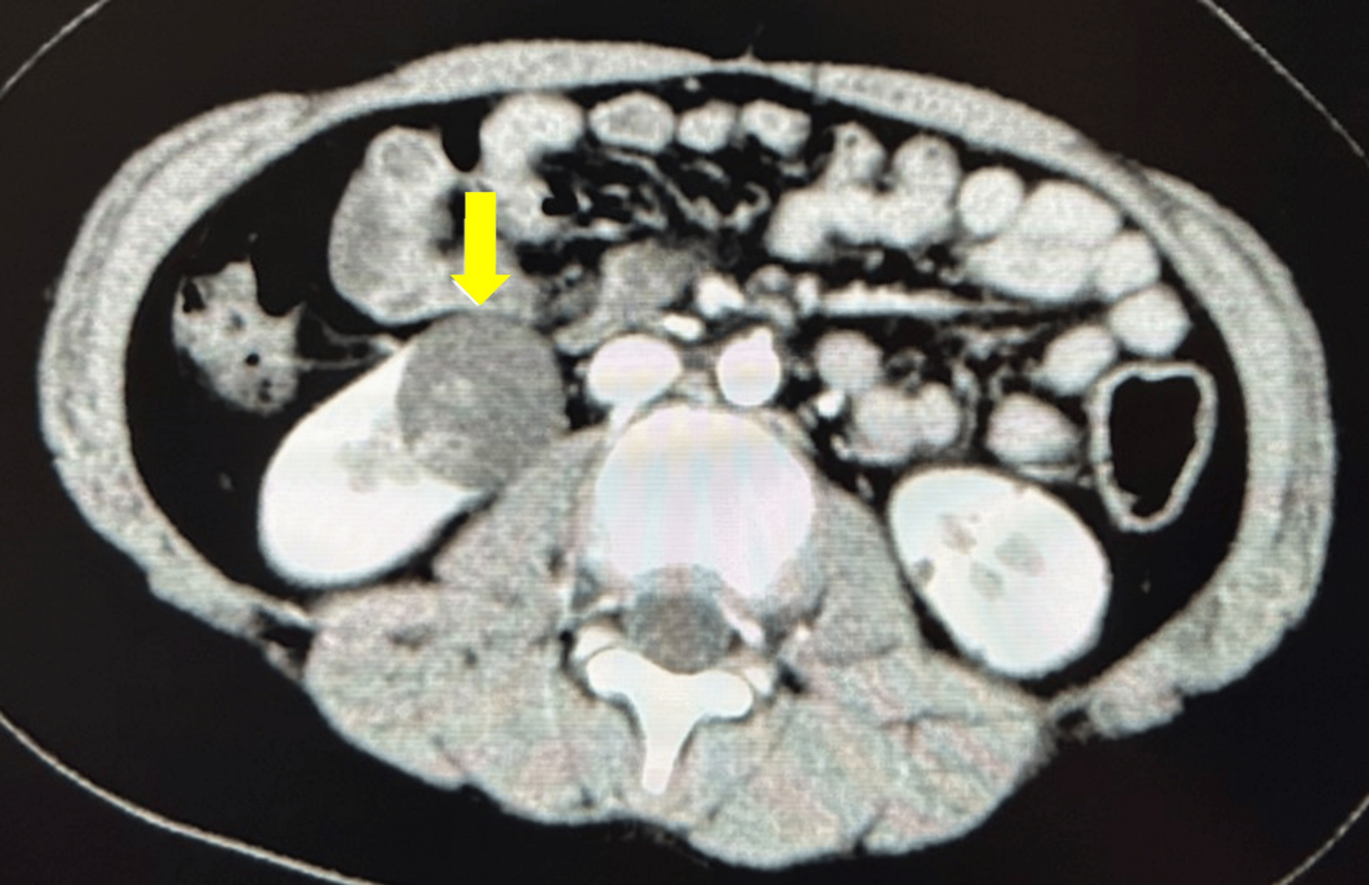
Contrast-enhanced CT Contrast-enhanced CT showing a cystic lesion (32 mm) with an enhancing solid component (16 × 12 mm) suggestive of malignancy.

The patient is an 11-year-old boy, 139.6 cm tall and weighing 49.5 kg, who was diagnosed with anaplastic glioma at two years of age. He underwent surgical resection, followed by adjuvant radiotherapy and chemotherapy with temozolomide, and has remained recurrence-free to date. He had no comorbidities and no family history of malignancy. The patient presented with a gradually enlarging cystic lesion in the right kidney that had been under observation. However, due to its growth and the presence of a solid component within the cyst on contrast-enhanced CT, he was referred for surgical management on suspicion of cystic renal carcinoma. Contrast-enhanced computed tomography (CT) (Figure 1) revealed a 32-mm cystic renal mass containing a 16 × 12 mm enhancing solid component suggestive of malignancy.

Surgical procedure

**Figure 2:**
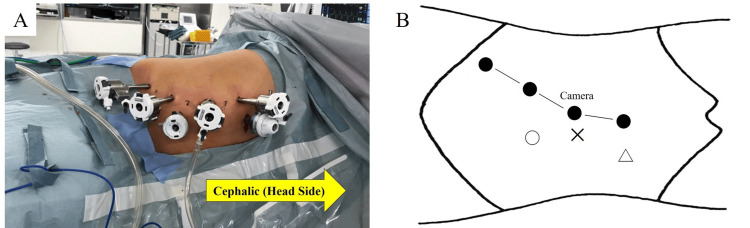
Patient positioning and port placement (A) The patient is placed in a mild Trendelenburg position. (B) Port layout: ● 8-mm robotic ports (camera and robotic arms), 〇 12-mm assistant port, △ 12-mm assistant port (AirSeal™ system), with approximately 5-cm spacing between ports. Image credits: Takayuki Ohzeki.

RAPN was performed using the da Vinci Xi surgical system (Intuitive Surgical, Sunnyvale, CA, USA). A transperitoneal approach was chosen. The patient was positioned in a mild Trendelenburg position (Figure 2A), and four robotic ports along with two assistant ports were placed (Figure 2B). Operative time was 189 minutes, with console time of 119 minutes and renal artery clamping for 21 minutes. Estimated blood loss was 77 mL. The mass was excised intact without rupture, and renal parenchymal preservation was adequate. No intraoperative complications were noted.

The patient recovered uneventfully and was discharged on postoperative day 6. At the 24-month follow-up, there was no evidence of recurrence or metastasis.

Pathological findings

**Figure 3:**
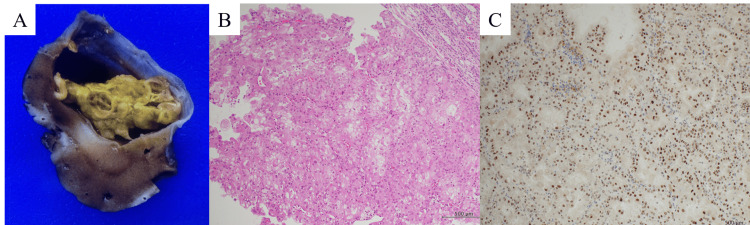
Pathological findings A. Gross specimen: 2.5 × 3.5 × 3.0 cm hemorrhagic cyst containing yellow papillary material. B. Hematoxylin & eosin staining (×200): atypical eosinophilic epithelial cells with papillary/microcystic architecture. C. TFE3 immunostaining (×400): positive nuclear staining confirming TFE3 rearrangement.

Gross examination of the specimen revealed a 2.5 × 3.5 × 3.0 cm hemorrhagic cyst containing yellowish papillary structures (Figure 3A). Histological evaluation showed atypical epithelial cells with eosinophilic cytoplasm, enlarged nuclei, and papillary to microcystic architecture within a vascular stroma (Figure 3B). Immunohistochemistry revealed positivity for CK20, AE1/AE3 (partial), Vimentin, P504S (AMACR) (partial), and TFE3 (Figure 3C), with negative staining for CK7, CD10, MelanA, HMB45, and c-kit. Although TFE3 split FISH (fluorescence in situ hybridization) was negative, diagnosis of tRCC was established based on morphology and immunohistochemistry.

## Discussion

tRCC is a rare entity defined by gene translocation involving the Xp11.2 locus. These tumors are known for their distinct histological appearance and immunohistochemical profile, particularly strong nuclear TFE3 expression [1-3]. Despite advances in molecular diagnostics, the interpretation of TFE3 split FISH can be challenging, particularly in the setting of cryptic intrachromosomal inversions, which may lead to false-negative results [9]. In this case, the patient developed anaplastic ganglioglioma at the age of 2 and subsequently tRCC at the age of 11. The occurrence of two distinct tumors at such a young age raises the possibility of an underlying cancer predisposition syndrome. Fukushima et al. reported a case of a boy who developed a brain tumor at the age of 2 and a renal tumor at the age of 11, in whom SMARCB1 mutations were identified in both tumors. The patient was diagnosed with rhabdoid tumor predisposition syndrome, an autosomal dominant hereditary condition [10]. Familial cases involving both atypical teratoid/rhabdoid tumor (AT/RT) and renal rhabdoid tumor due to the same INI1 (SMARCB1) gene alteration have also been reported [11], further supporting the role of genetic background in tumor predisposition syndromes. Although the possibility of other tumor predisposition syndromes, such as Li-Fraumeni syndrome, cannot be excluded, there was no significant family history. Rare cases of concurrent brain and renal tumors in childhood have been reported in the literature, suggesting the importance of long-term follow-up and genetic counseling in such cases.

Owing to its rarity, there is no established standard treatment protocol for tRCC, and therapeutic strategies are often extrapolated from adult RCC guidelines [4,5,12]. Complete surgical resection remains the primary treatment modality for localized disease. RAPN offers a minimally invasive alternative, with advantages including reduced blood loss, shorter hospitalization, and nephron preservation [8].

While robotic surgery in pediatric patients has traditionally been limited to benign urologic conditions, its application is increasingly extending to pediatric renal malignancies. A recent systematic review identified 21 studies reporting a total of 20 pediatric RAPN cases [13]. Among these, only one case of tRCC treated with RAPN has been documented by Antar et al. [14]. Our case, thus, represents the 21st reported pediatric RAPN and the second documented case of tRCC managed with this approach, further supporting its feasibility in selected pediatric patients.

RAPN is well established in adult patients; however, its use in children is constrained by technical challenges related to the limited intracorporeal working space. In this case, an 11-year-old boy measuring 139.6 cm in height underwent RAPN via a transperitoneal approach, which was selected to provide a larger working space. Four ports were used, and the distance between the 8-mm ports was set at 5 cm to accommodate the patient’s small body size. This port configuration allowed for a surgical approach comparable to that used in adults. The cystic renal tumor was successfully excised without rupture, and adequate preservation of the renal parenchyma was achieved. To our knowledge, the youngest patient reported to undergo RAPN was two years old [13]. Based on our experience, RAPN can be safely performed in pediatric patients, provided that at least three ports can be adequately positioned, conferring benefits comparable to those observed in adult populations.

Although the use of adult-sized robotic instruments may restrict the broader application of RAPN in smaller children, this case demonstrates that, with careful port placement and meticulous surgical technique, RAPN can be safely executed in pediatric patients of suitable body size. Nonetheless, the long-term oncologic outcomes of RAPN for tRCC remain unknown and warrant further investigation through multicenter collaborative studies.

Several challenges persist in applying robot-assisted surgery for pediatric RCC. The limited number of reported cases precludes definitive conclusions regarding the safety and efficacy of this approach. Additionally, anatomical differences inherent to pediatric patients, along with tumor size and location, may limit the advantages of robotic surgery compared to conventional open techniques in certain scenarios. Thus, careful consideration of surgical indications and tailored selection of the surgical approach based on individual patient and tumor characteristics are paramount.

Building on this case, further accumulation of pediatric RAPN cases is necessary. Concurrently, research aimed at standardizing surgical indications and postoperative follow-up protocols should be pursued. Moreover, additional studies are needed to evaluate the efficacy of robot-assisted surgery in other pediatric renal tumors and in patients with comorbid conditions.

It has been reported that tRCC tends to carry a high risk of recurrence and poor prognosis in advanced cases [4]. Given that the patient in this case was only 11 years old, long-term surveillance and continuous follow-up are considered essential.

## Conclusions

This case highlights the feasibility and potential of RAPN in treating pediatric RCC, including rare subtypes such as tRCC. Successful application of RAPN requires careful patient selection, preoperative planning, and consideration of technical limitations. The integration of minimally invasive surgery into pediatric oncology practice should proceed cautiously, with emphasis on accumulating long-term oncologic data and establishing guidelines for its safe implementation in malignancies traditionally managed with open surgery. As robotic technology continues to evolve, it may offer increasingly viable options for nephron-sparing surgery in selected pediatric cases.
